# Implications of Caspase 1/ Interleukin-1 Beta (IL-1β) Signaling and Hypoxia-Inducible Factor 1-Alpha (HIF-1α) on Diabetic Retinopathy Pathology

**DOI:** 10.7759/cureus.42479

**Published:** 2023-07-26

**Authors:** Drew Garber, Shu Zhu

**Affiliations:** 1 Osteopathic Medicine, Philadelphia College of Osteopathic Medicine, Suwanee, USA; 2 Physiology, Pharmacology, Renal Medicine, Cardiology, Philadelphia College of Osteopathic Medicine, Suwanee, USA

**Keywords:** signal transduction, retina, oxidative stress, neovascularization, hif1-a, caspase-1/il-1b, vegf-a, diabetic retinopathy

## Abstract

Diabetic retinopathy (DR) is the leading cause of adult blindness and partial vision loss in modern society for hyperglycemic patients. Accordingly, new treatment options are imperative to the overall reduction of DR prevalence and the ongoing progression of already affected candidates. There are many diseases that are the direct result of specific inflammatory processes. In this literature, DR is looked at as a potential disease that can be alleviated by targeting caspase 1/ interleukin-1 beta (IL-1β), and hypoxia-inducible factor 1-alpha (HIF-1α) signaling pathways and reducing cytokine mobilization within retinal tissues. Caspase-1 is thought to be upregulated during retinal capillary degeneration and other ocular complications. Hypoxia-inducible factor 1-alpha (HIF-1α) is implicated in its role in neovascularization and cell apoptosis within a retinal cell line. Both of these proteins are shown to be significantly elevated in hyperglycemic and galactosemic mice and, when knocked out, seem to have the reverse effect, showing that there is room for potential non-invasive therapy involving these proteins in the future. Vascular endothelial growth factor-alpha (VEGF-A) is also examined as a main signaling protein involved in the manifestation of DR.

## Introduction and background

Diabetic retinopathy (DR) is one of the most deteriorating secondary manifestations of clinical hyperglycemia in modern-day society. With the significant increase in type 2 diabetes patients, secondary pathological manifestations are associated with hyperglycemia and insulin resistance; these range from mild symptoms such as weight gain and sleep apnea to disabling conditions like peripheral neuropathy and diabetic retinopathy. Approximately 29 million Americans suffer from type 2 diabetes, with nearly a quarter of the population showing symptomology consistent with diabetic retinopathy [1]. Even with current treatment protocols to prevent the onset of DR, it remains one of the leading causes of adult blindness worldwide. Reports have shown that nearly 27% of all cases seen by an ophthalmologist daily include patients with DR, and this trend is not expected to slow down as rates of diabetes mellitus are exponentially increasing [1].

The implicated anatomy of DR includes the retina, which is specialized neural tissue in the back of the eye; it converts images the eye sees into specific nerve signals that the brain can interpret. When the blood vessels of the retina are compromised with severe anatomical damage, they become blocked, which cuts off most of the retina's blood supply [2]. This reduction of blood flow can cause other, new and fragile blood vessels to form, leading to optical pathologies such as blurry vision, floaters, and complete blindness [2]. These new blood vessels can swell and become leaky, creating scar tissue that was not already there. Due to neovascularization, most of these new blood vessels are very harmful to the overall functioning of the retina. Diabetic retinopathy can cause secondary complications such as glaucoma, vitreous hemorrhage, and even total detachment of the retina [2]. A pertinent concern for DR is the potential for rapid onset and quick progression of the disease into more severe pathologies. For instance, there are mild non-proliferative, moderate non-proliferative, severe non-proliferative, and proliferative retinopathy, each carrying its own unique symptomology and treatment protocols [3]. At the mild non-proliferative stage, there is an elevated risk that vision can be damaged, but significant bleeding and the buildup of scar tissue are yet to be seen [3]. Typically, in this stage, there is no need for immediate laser or anti-vascular endothelial growth factor (VEGF) treatment, but it is advised to have consistent screening appointments every four to five months [3]. The diagnosis of proliferative diabetic retinopathy should be taken very seriously, as it comes with many health concerns, such as macular edema, besides the potential risk of hemorrhage [3]. There are also increased levels of neovascularization because the blood within the retinal chamber becomes very viscous, leading to circulation and perfusion issues [2]. The formation of these underdeveloped blood vessels lacks many of the nutrients required to survive. Without the proper nutrients, there is a much greater chance that a person will develop proliferative retinopathy and have to resort to a more invasive approach. This is mainly due to growth factors released on the basis of sufficient retinal ischemia and hypoxia [4]. Once growth factors like insulin-like growth factor (IGF-1) and VEGF-A are released from the retina, neovascular activity increases and starts the vicious cycle of generating new, underdeveloped capillaries in the retinal vasculature [4]. It has been shown in previous studies that scientists have tried developing a somatostatin treatment that directly works by inhibiting IGF-1's activity but was ultimately unsuccessful [4].

## Review

Methodology

Literature was reviewed from common science libraries PubMed and Embase using various combinations of the search terms “diabetic retinopathy,” “HIF-1a,” “caspase-1,” and “IL-1B”. Studies were included if they discussed the implications of these signaling pathways in DR clinically or scientifically. Studies were excluded if they were not in the English language. A total of 37 studies were identified.

Current treatment of diabetic retinopathy

The treatment of DR includes pan-retinal photocoagulation, vitrectomy, anti-VEGF medication, and overall control of hyperglycemia [5]. When a patient is suspected of having DR, ophthalmologists may perform angiography to assess the retinal vasculature [3]. One common treatment modality is pan-retinal photocoagulation [6]. This form of photocoagulation obliterates the new leaky blood vessels formed due to neovascularization [7]. This procedure can also help destroy necrotic or abnormal tissue in the eye [7]. Most patients receiving this treatment have advanced proliferative DR, and the physicians believe there is no alternative to adopt [8]. This procedure is repeated at each patient visit to treat newly proliferated blood vessels and prevent the recurrence of previously treated vessels [8].

Proper glucose and insulin homeostasis should be the primary concern for anyone diagnosed with proliferative diabetic retinopathy (PDR) or non-proliferative diabetic retinopathy (NPDR) [9]. In many cases, maintenance of blood glucose levels reduces the progression of disease and minimizes vision problems [9]. In the Action to Control Cardiovascular Risk in Diabetes (ACCORD) trial, patients who received extensive blood sugar control with a mean target hemoglobin A1c (HbA1c) level of under 6.0% resulted in a significantly decreased rate of progression of their DR symptoms when compared to those patients with a target HbA1c average of 7%-8% [10]. Furthermore, rapid fluctuations in glucose levels and a loss of overall metabolic control are key contributors to the onset and progression of DR [9]. Sudden changes in glycemic control cause damage to neuro-retinal cells, commonly seen in type 1 diabetics [10]. Glycemic variability tests (GV) are becoming the preferred tests because they are the major predictor of DR in patients who may be asymptomatic or at risk of developing PDR [9].

For patients with a mild stage of non-proliferative DR, surgical vitrectomy is a viable option [11]. Surgical vitrectomy entails the removal of the vitreous gel and prevents further hemorrhage or scar tissue formation [11]. The main goal of this procedure is to repair retinal attachments and prevent further macular degeneration [11]. There have been concerns about DR-specific vitrectomies due to the possibility of serious secondary complications from the procedure. In one study assessing the efficacy of DR vitrectomy, Constantin et al. concluded that 17%-26% of patients have reoccurring hemorrhage, cataracts, and neovascular glaucoma [11].

The advent of anti-VEGF treatment addresses DR on a mechanistic basis [12]. Vascular endothelial growth factor (VEGF) has been heavily implicated in the pathogenesis and overall development of DR. It is an angiogenic signaling protein that enables the formation of blood vessels [12]. In response to hypoxia, hypoxia-inducible factors (HIF) are stimulated within local tissues, leading to the production of VEGF-A. Receptor binding of VEGF-A, an endogenous circulating ligand for VEGF receptors, leads to the activation of tyrosine kinases, thereby stimulating angiogenesis [12]. Thus, VEGF-A inhibitors are effective in ameliorating and potentially reversing some aspects of DR. Moreover, VEGF-A inhibitors may reverse the side effects of other treatments, such as pan-retinal photocoagulation. Some of these drugs include ranibizumab, bevacizumab, and aflibercept [12]. In current practice, the protocol is initial treatment with anti-VEGF injections and secondary treatment with photocoagulation [12]. Other secondary components of VEGF upregulation, such as growth factors like insulin and IGF-1, are important to take into consideration.

Neovascularization, macular edema, and retinal hemorrhage

There are three hallmark clinical manifestations of DR: retinal angiogenesis, diabetic macular edema (DME), and ocular hemorrhage [13]. Understanding which stage and specific symptom a patient presents with is the basis of a personalized approach to DR management [13]. Similarly, this reveals distinct molecular pathophysiologies of PDR and NPDR [13]. More specifically, the presence of cotton wool spots may reveal activation of the HIF-1α hypoxic response pathway [14]. Hypoxia-inducible factor 1-alpha (HIF1-α) mediates the primary hypoxic responses within the retina, and it can be targeted to reduce the disease burden of DR [14]. Diabetic retinopathy and DME are interlinked in their molecular pathophysiology as they share many of the same biological pathways, including oxidative stress and inflammation [14]. Protein kinase C (PKC) is a kinase that controls various second messenger cascades [15]. In DR and DME, some data suggests blocking the beta subunit of PKC can slow progression by multiple mechanisms [15]. Blockade of PKC can alter smooth muscle contractility, basement membrane protein synthesis, angiogenesis, and endothelial permeability [15]. Additionally, PKC causes the release of cytokines, transforming growth factor-beta (TGF-β), and growth factors. Polyphenol compounds are under study as treatments for DR. Polyphenol rottlerin directly inhibits the PKC delta isoform and other enzymes like calmodulin and protein kinase A (PKA) [16]. Another therapeutic candidate, ruboxistaurin (RBX), blocks the beta subunit of protein kinase c, and in experimental trials, patients with DME successfully responded to RBX treatment [16]. To summarize, past and current drug treatments target varying intracellular signaling proteins to alleviate DME and DR potentially, but none have come without side effects that qualify for a successful therapy option [16].

Caspase-1 and IL-1β signaling

Caspases are cysteine proteases that respond to apoptotic stimuli; they function to cleave proteins such as precursors to inflammatory cytokines (interleukin-1 (IL-1), tumor necrosis factor-alpha (TNF-α)) into active forms [17]. Caspases have two main functions: the proteolytic activation and release of pro-inflammatory molecules and the activation of programmed cell death [17]. The caspase-1 family is unique in that it can perform both functions [17]. Caspase-1 is shown to directly interact with IL-18 (interferon-inducing factor), which is responsible for relieving inflammation in mammal models (adenylate kinase 1 (AK1)) [17]. Additionally, the caspase/IL-1β pathway is thought to directly impact the early development of retinal capillaries via IL-1β receptor activation [17]. Again proving that, under hypoxic conditions, HIF-α is activated, which ultimately leads to caspase-1 and an acute inflammatory response [17]. It is evident that in Müller cells, caspase-1, along with other hypoxic transcriptional factors, is activated under hyperglycemic conditions [17]. This could give further credence to the notion that knocking out this enzyme or downregulating its activity can alleviate DR pathology without administering invasive laser treatment. Interestingly, caspase-1 activity in retinal cells is increased in mouse models of galactosemia [17]. This model shows a similar onset of development for diabetic-like retinopathy pathology compared to regular hyperglycemic mice [17]. The advanced death of retinal capillary cells is a precursor to the progression of other severe lesions that model DR, suggesting that these caspases most likely have a significant impact on the severity of NPDR and PDR. Thus, caspase-1 and other caspase families are promising targets to understand the role of retinal apoptosis in DR. Furthermore, the mechanism by which galactosemic patients are affected by IL-1β could be different from that of hyperglycemic patients. There is evidence that blocking the caspase-1/IL-1β signaling cascade via antibiotics can lead to the rescue of DR pathology [17]. Minocycline and, in some cases, tetracycline have successfully been able to inhibit the caspase-to-IL-1β cascade after two months [17]. Intraperitoneal minocycline injection led to a reduction of caspase-1 expression and overall inflammation within the retinal capillaries of hyperglycemic mice [17]. Four months after treatment in this same cohort, neovascularization was inhibited via the inactivation of this signaling pathway [17]. These results shed light on the future treatment of DR. Antibiotics such as minocycline or other agents that block caspase-1/IL-1β can be used as non-invasive treatments [17].

Hypoxia-inducible factor 1-alpha (HIF-1α) and reactive oxygen species (ROS) impact on hyperglycemia conditions for DR candidates

Recent studies demonstrate that pro-inflammatory cytokines (IL-1b, IL-6, and TNF-α) in retinal tissues are involved in developing diabetic retinopathy [18]. One of the most critical proteins responsible for neovascularization is vascular endothelial growth factor (VEGF). Additionally, it has been shown that there is a strong correlation between the serum levels of HIF-1α in patients and the development of DR [18,19]. Hypoxia-inducible factor 1-alpha is important in regulating ischaemic hypoxia in DR, and hypoxia causes HIF-1α to eventually stimulate VEGF production, which thereby leads to angiogenesis and vasculogenesis [18,19]. Streptozotocin (STZ) is an antineoplastic agent that destroys beta cells in the islets of Langerhans, inducing hyperglycemia. Streptozotocin-induced hyperglycemia led to increased HIF-1α levels [18]. In these same rats, administration of 2-methoxyestradiol (2-MET), which inhibits the release of HIF-1α, led to a net decrease in pro-inflammatory cytokine levels within the retina in hyperglycemic mice treated with streptozotocin (STZ) [18]. Another notable finding in STZ-induced DR is that HIF‐1α leads to the upregulation of IL‐6 and TNF‐α receptors as well as caspase‐3, which is involved in consequent retinal damage in animals [18]. Hypoxia-inducible factor 1-alpha has been found to be activated by the nicotinamide adenine dinucleotide phosphate (NADPH) oxidase (NOX) pathway indirectly through proto-oncogene c-SRC (SRC) oncogenes [19]. The nicotinamide adenine dinucleotide phosphate (NADPH) oxidase functions within the electron transport chain (ETC) and releases reactive oxidative stress species. It is also implicated in host immune and pro-inflammatory responses [19]. The buildup of NOX typically leads to other secondary complications, such as cellular stress leading to different disease types [19]. When NOX is active, there is a buildup of reactive oxygen species in the cell, which, in turn, activates HIF-1α [19]. The activation of HIF-1α activates VEGF-A, leading to the angiogenesis characteristic of DR [18,19]. The nuclear factor kappa B (NF-Kb) pathway has also been implicated in the pathogenesis of DR. An increase in the phosphorylation of NF-Kb at its p-65 subunit led to the upregulation of NF-Kb expression and DR [20].

An increase in vascular permeability and blood-retinal barrier permeability can lead to macular edema as well as other secondary complications [19,20]. With neovascularization, there is an increased rate of vessel leakage and hemorrhage within the retina. It is evident that hypoxia in retinal capillaries is exacerbated in the presence of hyperglycemia. This causes a positive feedback loop that drives the pathogenesis of DR. Hypoxia-inducible factor 1-alpha is involved in cellular processes like energy metabolism, cell survival, and peripheral nerve regeneration, yet further research from the perspective of DR is necessary to assess its role in disease progression [18]. While studies have shed light on the role of HIF-1α in the molecular pathophysiology of DR, there are yet no translational advances related to blocking HIF-1α without side effects [18]. Knocking out this gene in humans is not viable as it is a master regulator and main activator of over 45 genes, including glucose transport and glycolytic enzymes [18]. A future goal for this area of research should be to specifically target retinal cells to adapt to hypoxic conditions to properly sustain biological homeostasis in DR patients.

Vascular endothelial growth factor-alpha (VEGF-A)-induced retinal inflammation

Vascular endothelial growth factor-alpha (VEGF-A), originally termed "tumor angiogenesis factor" by Judah Folkman, is a signaling protein that stimulates the growth and development of novel blood vessels [21]. It is a subfamily of growth factors involved in vasculogenesis or the de novo formation of the embryonic circulatory system [21]. It is part of the system that replenishes the oxygen supply to tissues when blood circulation is hindered [21], can act at several levels of the vessel beds, keeping endothelial cells alive while increasing microvascular permeability, and can cause vasodilation [4,21]. It is also important in the kidney, where it regulates renal glomerular capillary function and glomerulogenesis [21]. It even plays a large role in the musculoskeletal system, providing a means for regeneration, endochondral bone formation, and cardiovascular physiology [2]. Vascular endothelial growth factor-alpha (VEGF-A) overexpression in humans contributes to many disease types, including retinal disease [22]. More specifically, VEGF-A can increase blood-retinal barrier permeability, leading to an increase in retinal interstitial fluid, typically resulting in DME. This is thought to arise from an increase in pro-inflammatory cytokines in the blood, which stimulates the release of macrophages and other cells to mobilize to the site of damage. Adenylate kinase 2 (AK2), an important implication of VEGF-A, includes the interaction between the retinal blood barrier and neovascularization [18]. Neovascularization is related to the breakdown of the retinal blood barrier, and neovascularization itself further propagates retrobulbar blocks (RBBs) breakdown in a positive feedback cycle. Hypoxia-inducible factor 1-alpha and VEGF-A are linked in the pathology of DR, as the upregulation of HIF-1α leads to the release of VEGF-A [23]. Cotton wool spots are the sequelae of retinal fluid buildup, resulting in hard exudate formation [2]. This hardened yellow exudate contributes to the symptomology like blind spots, blurry vision, and floaters that are often common in patients with DR. Mechanistically, VEGF-A induces vessel leakage in DR by the phosphorylation of retinal tight junctions, such as zona occludens [23]. The breakdown of these tight junctions is typically consistent with a deteriorating retina and an increase in endothelial cell permeability. In addition, drugs that prevent the breakdown of these tight junctions can improve patients with mild to moderate DR [21]. The specific mechanism by which this happens is circulating VEGF-A-mediated stimulation of tyrosine kinase, which stimulates angiogenesis [23]. Interestingly, patients with type 2 diabetes and NPDR have increased VEGF-A in the blood, which affects retinal Müller cells. This is crucial, as VEGF-A has specific effects on inflammatory pathways within the retina (AK3). In a murine model of STZ-induced hyperglycemia, it was shown that mice with induced diabetes have increased levels of VEGF-A and resulting angiogenesis [23]. The same experiment also found that HIF-1α levels were similarly affected by the induction of hyperglycemia via STZ [23]. Vascular endothelial growth factor-alpha has been implicated in releasing pro-inflammatory cytokines, monocytes, and macrophage expression [16,17]. After this initial response, leukocyte rolling and the stimulation of adhesion molecules happen simultaneously, further increasing vascular permeability [23]. Taken together, patients with DR typically have higher levels of the signaling protein VEGF-A, increasing retinal angiogenesis and worsening PDR.

During the earlier stages of DR, proteins such as intracellular adhesion molecule-1 (ICAM-1) and TNF-α are highly regulated [24]. Vascular endothelial growth factor-alpha increases the expression of ICAM-1 and other adhesion molecules in endothelial cell capillaries [18,24]. In addition to these two pro-inflammatory proteins, the transcription factor NF-Kb has been implicated in the development of DR [24]. This transcription factor is responsible for increased blood-retinal barrier permeability and can contribute to microvascular lesions and general macular edema [24]. When measuring the phosphorylation of the NF-Kb p-65 subunit in diabetic mice, it was found that there was a two-fold decrease in the density of phosphorylated p-65 [20,24]. Targeting this specific subunit in the future can potentially alleviate or even reverse the symptoms outlined above. The impact of VEGF-A in DR may be linked to a sequence variation in the VEGF gene [25]. In a study performed on 554 diabetic individuals, it was determined that there was a strong association between the haplotype TCC GCG and an increased risk of developing DR [25]. Subsequently, there can also be environmental changes that activate underlying predispositions to DR associated with this particular gene [25]. Several other VEGF-A single nucleotide polymorphisms (SNPs) are linked with an elevated risk of developing blinding DR in both type 1 and type 2 diabetes, isolated from the duration of diabetes and the severity of glycemic control [9,25]. These same variations can make it easier and more efficient for physicians to correctly diagnose a patient by using these specific VEGF-A biomarkers.

Adverse effects of anti-VEGF therapy

For most patients, the blockade of VEGF-A stands as the primary method of treatment for DR, but there is a percentage of patients who cannot respond or have a hyper-immune response to this treatment. Some consequences associated with blocking VEGF-A activity include hypertension, proteinuria, and impaired healing of wounds [22]. These three examples are mainly a result of systemic VEGF inhibition; other symptoms of chronic use of VEGF inhibitors include infertility, lack of vessel development, and inhibition of cardiac remodeling [20,26]. People with diabetes also have an increased risk of developing myocardial ischemia if VEGF levels are systemically low [26]. Besides the short-term side effects of intravitreal injection itself, there are other ocular complications that can arise from VEGF-A inhibition [27]. First, the secretion of VEGF is essential in the retinal epithelium for the survival of the choriocapillaris [22]. The choriocapillaris is the network of blood vessels underneath the retina and exhibits a neuroprotective effect within the retinal vasculature [22]. When blocking these cells with anti-VEGF treatment, inflammation is inhibited within capillaries, but some of that neuroprotective effect is lost [22]. There is a significant reduction in ganglion cells in mice when isoforms of VEGF-A are blocked in rats [22]. The creation of a therapeutic agent that will act as a mediator for angiogenesis while retaining all the inherent molecular benefits within this growth factor is a future goal. In addition to the side effects of anti-VEGF therapy, patient adherence, cost, and proper follow-up visits should all be noted as critical in initial treatment [28-30].

Oxidative stress and endocrine control in DR

Nicotinamide adenine dinucleotide phosphate (NADPH) oxidase (NOX) is a crucial enzyme in the activation of downstream pathways that lead to the buildup of reactive oxygen species (ROS) [19]. A key component in NOx signaling is the renin-angiotensin system, which is responsible for the critical hormone aldosterone [27]. Aldosterone is mainly implicated in the renin-angiotensin system (RAS) by increasing the amount of sodium in the blood, thereby increasing blood pressure and the amount of potassium in the urine. (KAB4) hypertension is also thought to be responsible for furthering DR complications by leading to microvascular vessel damage [27]. Nuclear factor kappa (NF-Kb) and RAS pathway activation in the retina led to inflammation by up-regulation of ICAM-1 and VEGF, which are relieved by an angiotensin receptor blocker [27,31]. The renin-angiotensin system (RAS) may have a crucial role in the pathogenesis of DR, and the role of RAS inhibitors to alleviate DR is being investigated [27,31]. Currently, the exact source of ROS buildup within the retina is being examined, but it is thought to stem from the glycolytic pathway, thereby increasing NADH and tissue lactate/pyruvate ratios [32]. There is also stimulation of the tricarboxylic acid cycle flux, which results in the buildup of electrons within the mitochondria, producing more ROS [32]. Though there seems to be a direct relationship between diabetes and the buildup of ROS in DR patients, it is still unclear how these two are interlinked. The mitochondria are thought to be critical in increasing ROS levels, but this is still being studied [32]. Oxidative stress damages protein conformation, lipid structure, and general macromolecule function [31]. A possible regimen of sufficient vitamin E intake (if there is a deficiency) has shown positive anti-oxidant effects, leading to a sharp reduction in ROS species [32]. Vitamin E also acts as a neuroprotectant and has antiangiogenic characteristics [32]. Compared to other tissue types, retinal tissue has a substantial oxygen uptake capacity and higher glucose oxidation levels, in turn creating a susceptible environment in which ROS buildup is more common [32]. Oxidative stress also has a clear connection to the reversal of DR pathologies, as it is lowered when glycemic levels are controlled [33]. Hydrogen peroxide (H2O2) content and membrane lipid peroxidation are also higher in the retina vasculature of diabetic mouse models and humans [33,34]. Because oxidative stress displays a disparity between the formation and hindered removal of oxidative species, the anti-oxidant defense system of the cell is a pivotal piece in the cumulative oxidative stress experienced by a cell [33].

Future direction and implications

In the near future, effective non-invasive therapies need to be developed to improve the management of DR. Anti-VEGF-A treatment paired with steroid injections and pan-retinal photocoagulation is the current preferred method to eliminate the majority of angiogenesis in the retina [12,22,33]. Further research is needed on specific transcription factors that have an epigenetic effect on the pathophysiology of DR. Another crucial question that needs to be answered is how the presence of VEGF-A specifically impacts caspase 1, IL-1B, and HIF-1α signaling indirectly within retinal capillaries [24]. Another pertinent question that needs to be addressed is whether sufficient glycemic control reduces VEGF-A levels. A regimen that consists of a balanced diet and strenuous exercise can reverse some of the pathologies associated with DR by simply downregulating different inflammatory pathways in the blood [35]. Anti-inflammatory polyphenols, like resveratrol, could provide relief for patients. Dong et al. showed that resveratrol-coated intravenously (IV)-injected gold nanoparticles (AuNPs) in STZ-induced diabetic rats for three months provided protection against DR [36]. Next, it is paramount to correctly pinpoint which specific pathways are turned on in the mitochondria, ultimately leading to electron overload and ROS buildup; ROS is the primary precursor for other damage caused to retinal capillaries and the microvasculature [19,31]. Another area of study linked to DR that shows promising results is the activation and overexpression of different inflammatory receptors like toll-like receptor-2 (TLR-2) and TLR-4 [37]. These receptors are responsible for the overall activity of microvascular endothelial cells [37]. Genetic deficiency of these toll-like receptors has been shown to alleviate some symptoms associated with diabetic nephropathy [37]. Knocking out TLR-2 and TLR-4 has also been shown to attenuate some of the adverse effects of high glucose levels [37]. These effects include hypertension, oxidative stress, decreased blood vessel elasticity, and angiogenesis. To confirm that these toll-like receptors substantially impact DR pathology, it was tested whether high glucose-induced ROS increased TLR-4 expression [37]. It was found that this was indeed the case and confirmed the original hypothesis that increased blood pressure leads to the buildup of ROS, which, in turn, increases inflammatory biomarker expression [19,30,37]. A deeper molecular characterization of the pathophysiology of disease progression in DR would allow for personalized care with targeted therapies. When really looking at the epidemiology and outcomes of DR, there are commonalities that can be attacked from different angles. For instance, significant risk factors aside from hyperglycemia include hyperlipidemia and nephropathy [2]. Another significant risk factor is the actual duration of one's diabetic condition and the degree of Hba1c elevation [9]. This condition has great biovariation, further necessitating personalized care. If it is possible to isolate the original causes of DR when taking into account all of these variables, it will be much easier to find the right therapy.

## Conclusions

The pathways involving caspase-1/IL-1β, VEGF-A, and HIF-1α hold significant promise for addressing DR. Caspase-1, which induces the pro-inflammatory cytokine IL-1β, has shown substantial upregulation in hyperglycemic patients. Inhibition of caspase-1 using antibiotics, such as minocycline and tetracycline, has demonstrated potential in retinal cell lines. Gene therapy utilizing ribonucleic acid (RNA) interference to modulate caspase-1 expression in retinal cells is a potential treatment option in this patient population. Another crucial transcription factor in DR is HIF-1α, which is associated with angiogenesis in DR patients and responds to nutrient deprivation and hypoxia. Reducing HIF-1α leads to a decrease in VEGF-A expression, providing promising evidence for mitigating retinal bleeding or vessel leakage. Studies using HIF-1α knockout models have shown relief from retinal leakage, inflammation, and neovascularization. Developing HIF-1α inhibitors, such as monoclonal antibodies that directly target and inhibit VEGF-A activity, holds promise in the management of DR. Investigating sustained-release drug delivery systems for controlled administration of VEGF-A inhibitors is another targeted approach for DR. While further research is needed to elucidate the exact mechanism, blocking the expression of ICAM-1, a cell adhesion molecule, appears to decrease chronic retinal inflammation. Furthermore, the accumulation of ROS due to NADPH oxidase activation and the subsequent phosphorylation of the p-65 subunit of NF-Kb play a significant role in angiogenesis and blood-retinal barrier breakdown. Considering these findings, targeting these pathways and molecules holds potential for the development of effective therapies to address DR. Further investigation and understanding of the mechanisms involved are imperative for advancing treatments in this field.
